# Improving Nanosilica Fluidization by Premixing with Geldart A and B Particles: A Detailed Region-Wise Study

**DOI:** 10.3390/nano15110822

**Published:** 2025-05-29

**Authors:** Syed Sadiq Ali, SK Safdar Hossain, Mohammad E. Ali Mohsin, Mohammad Asif

**Affiliations:** 1 Department of Chemical Engineering, King Faisal University, P.O. Box 380, Al-Ahsa 31982, Saudi Arabia; snooruddin@kfu.edu.sa (S.S.H.); maa.ali@kfu.edu.sa (M.E.A.M.); 2 Department of Chemical Engineering, King Saud University, P.O. Box 800, Riyadh 11421, Saudi Arabia; masif@ksu.edu.sa

**Keywords:** nanosilica, fluidization, hydrodynamics, assistance, particle premixing, Geldart group, contraction

## Abstract

Ultrafine nanosilica exhibits a strong tendency to form agglomerates, with sizes often several orders of magnitude larger than the primary particles. This agglomeration severely impairs its effectiveness in fluidization and other applications requiring uniform powder dispersion. To address this issue, the present study employed an assisted fluidization technique involving premixing of nanosilica with small amounts of external inert particles. The aim was to disrupt the structural integrity of the agglomerates by altering the inter-agglomerate force equilibrium. Two types of inert silica (SiO_2_) particles, representing Geldart groups A (finer) and B (coarser), were individually premixed with the nanosilica in different proportions. This strategy led to a significant reduction in both the minimum fluidization velocity ($U_{mf}$) and fluidization hysteresis. Moreover, a clear vertical segregation pattern emerged within the fluidized bed: the finer Group A particles (S-A) primarily enhanced fluidization in the upper and middle regions, while the coarser Group B particles (S-B) were more effective in the middle and lower regions. Interestingly, even at low premixing proportions, a significant volumetric contraction (up to 40%) of the premixed nanosilica bed was observed, which confirmed the disruption of the inter-agglomerate force balance within the nanosilica, contributing to enhanced fluidization behavior.

## 1. Introduction

Nanoparticles are highly attractive for a wide range of applications due to their exceptionally high surface area-to-volume ratio, which results from their ultrafine size. However, their pronounced tendency to agglomerate, driven by strong interparticle forces (IPFs), significantly limits their effectiveness in most applications. Therefore, overcoming these IPFs to achieve effective deagglomeration remains a major challenge in realizing the full functional potential of nanoparticles, particularly in large-scale industrial applications [1,2,3,4,5,6,7].

Fluidized beds are widely utilized in the chemical and petrochemical industries due to their excellent gas–solid mixing, improved interfacial contact, low pressure drop, and high heat and mass transfer rates [8,9,10]. However, the hydrodynamic behavior and overall performance of fluidized beds are highly sensitive to the physical properties of the solid particles, particularly their size and density [11,12].

Geldart [13] classified solid particles into four distinct groups—A, B, C, and D—based on their size and density. Group A particles typically range from 30 to 100 μm in diameter; they are easily aeratable and exhibit smooth fluidization with uniform bed expansion. Group B particles are larger, typically ranging from 100 to 1000 μm and are characterized by bubbling fluidization with limited bed expansion and non-uniform flow behavior. Of particular relevance to this study are Group C particles, which are extremely fine, with diameters less than 30 μm [14,15,16]. These particles are highly cohesive, resulting in poor fluidization quality and a non-homogeneous bed structure [17,18]. Ali et al. [19] investigated the behavior of ultrafine powders in fluidized beds and identified three distinct vertical regions based on agglomerate size. The upper region contained fine agglomerates approximately 10 times smaller than the large, rigid agglomerates found in the bottom region, which had an average diameter of about 150 μm. The middle region consisted of agglomerates of varying sizes, generally averaging about half the size of those in the lower section. Similarly, Zhao et al. [20] reported that in fluidized beds of nanosilica, the bottom region contained agglomerates with an average size of 280 μm, while the upper region was predominantly composed of finer particles.

The fluidization of ultrafine powders often requires additional energy to overcome strong interparticle forces (IPFs) [21,22]. This energy augmentation can be delivered through various internal or external excitation methods, including acoustic vibrations [23,24,25,26], oscillating particles under a magnetic field [27,28] mechanically vibrated beds [29,30,31,32], shear mixers [33] or ultrasonic comminution devices [34]. However, these methods typically involve significant equipment modification in addition to being energy intensive. In contrast, certain alternative techniques, such as flow pulsation [35,36,37,38,39,40] and particle premixing [41,42,43,44,45], offer simpler, more practical solutions. These methods are easier to implement, require minimal system modifications, and function effectively without the need for additional energy input.

In the present study, a particle premixing technique is employed, wherein inert particles were selected based on their physical properties and premixed with the primary particles within the fluidized bed. The mutual interaction between the inert and primary particles alters the interparticle force balance among the primary particles, thereby promoting deagglomeration and enhancing the hydrodynamic behavior of the bed. Song et al. [46] premixed Geldart Group A coarse particles (65–120 µm) with SiO_2_ and TiO_2_ nanoparticles in proportions ranging from 10 to 40 wt%. Their results indicated a marked improvement in fluidization quality, along with a significant reduction in particle elutriation. Moreover, bed expansion, terminal velocity, and the expansion index—calculated using the Richardson–Zaki equation—increased with an increase in premixing. Similarly, Duan et al. [47] reported improved fluidization quality of SiO_2_ and ZnO nanoparticles upon premixing with relatively large proportions (15–45%) of Group A external particles. However, the coarse Group B particles (200–1000 μm) yielded less favorable results, with an apparent increase in $U_{mf}$ and bed non-homogeneity at higher premixing ratios. These results suggest that at higher proportions, the fluidized bed behavior becomes increasingly dominated by the characteristics of the coarser external particles. In contrast, premixing small proportions of external particles (4.5 vol% and 8.6 vol%) with SiO_2_ nanoparticles was found to significantly suppress the hysteresis phenomenon and substantially reduce the $U_{mf}$ [48]. Nonetheless, fluidization behavior was found to be largely insensitive to further increases in the proportion of external particles. In a related study, even a very small premixing proportion of 2.3 vol% was sufficient to eliminate the hysteresis and improve bed homogeneity. Increasing the premixing ratio led to a reduction in agglomerate size reduction and a notable contraction in bed volume [49]. In bed collapse studies of premixed fluidized beds, the addition of 2.3 vol% of Group A particles significantly improved collapse dynamics; however higher premixing levels (4.5% and 8.6%) did not yield any tangible benefit over the lower ratio [50].

Clearly, the appropriate selection of particles is a critical prerequisite for the successful implementation of particle premixing as an assisted-fluidization technique. In this study, we aimed to investigate the effect of premixing Geldart Group A and Group B particles on the fluidization hydrodynamics of a nanosilica bed. This selection was motivated by the contrasting fluidization characteristics of the two groups: Geldart Group A particles exhibit smooth particulate fluidization and possess a size distribution significantly overlapping with that of nanosilica agglomerates, while Group B particles are much coarser, typically exhibiting bubbling fluidization with an average size nearly 10 times larger than the nano-agglomerates. The use of differently sized particles for premixing in our study results in significantly different force–balance equilibria involving gravity, buoyancy, drag, and van der Waals forces. For instance, the terminal settling velocity of the S-A particles, calculated using Stokes’ law (valid for ${Re}_{p}<1$) under ambient conditions, is approximately 0.256 m/s. In contrast, the S-B particles, due to their larger size, exhibit a terminal settling velocity of about 1.6 m/s, as estimated using the Schiller–Naumann correlation. This demonstrates that a four-fold increase in particle diameter leads to more than a six-fold increase in terminal settling velocity. This phenomenon also justifies the size-based stratification of nano-agglomerates along the height of the bed in multiple regions.

Moreover, the premixing levels were kept low (<10%) to prevent external particles from dominating the fluidization behavior of resident nanosilica particles. Since vertical segregation of external particles often occurs along bed height, local variations in the bed hydrodynamics were carefully monitored by recording local pressure transients at a high frequency (100 Hz). In addition, the volume change of mixing, an important yet often overlooked aspect in mixed bed studies, was also investigated. This aspect was particularly important in the present context, considering the exceptionally high void fraction (~0.97) of the nanosilica bed.

## 2. Experimental

### 2.1. Set-Up

**Figure 1:**
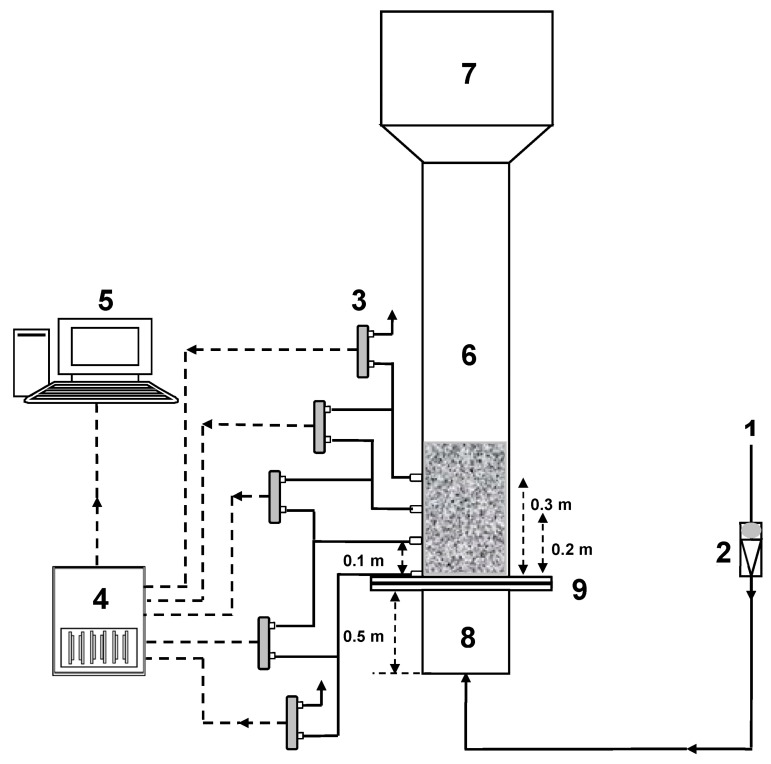
Schematic of the experimental set-up: (1) compressed air; (2) flow meter; (3) pressure transducer; (4) data acquisition system; (5) computer; (6) test section; (7) disengagement section; (8) calming section; (9) distributor.

Figure 1 illustrates the schematic of the set-up used in this work. The test section comprises of a transparent perspex column of an 0.07 m internal diameter and a 1.5 m height. A calming section of length 0.5 m was fitted before the test section to mitigate the end effects and turbulence in the entering fluid, while a disengagement section was fitted at the top of the test section to collect the elutriating particles from the column. Also, a distributor, with square pitch perforations and 4% fractional open area, was fitted at the entry of the test section, which ensured that the fluid is evenly distributed throughout the cross-sectional area of the test section. The fluid used to fluidize the bed is compressed air at ambient conditions. The fluid flow was set using two different ranged Gilmont flow meters.

### 2.2. Pressure Drop Measurements

The pressure drop was measured at four different bed regions in order to obtain a thorough insight into the bed. Also, the overall pressure drop transients were recorded during the experiments. The pressure tap positions are mentioned in Table 1. The extremely fast-response bidirectional differential pressure transducers (Omega PX163-005BD5V, Biel, Switzerland) were used to measure the pressure transients at a rate of 100 data/s in the form of voltage signals, which were recorded using a data acquisition system (National Instruments USB-6289, Austin, TX, USA) controlled using LabView software.

### 2.3. Solid Particles

**Figure 2:**
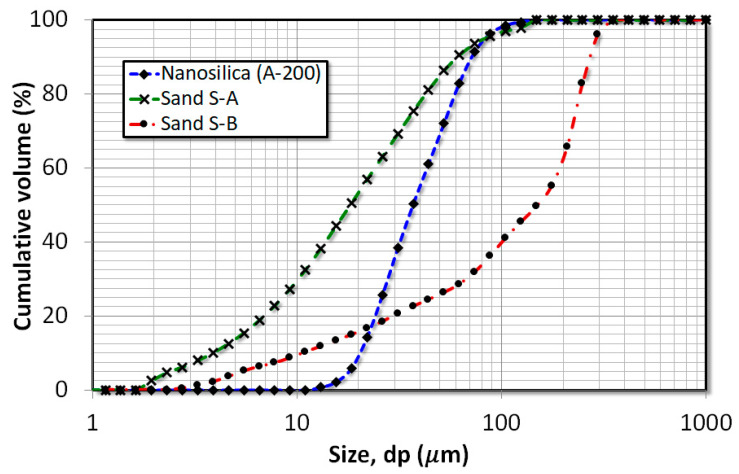
Size analysis of nanosilica and external particles used.

The nanosilica is used as the primary nanoparticle in the present work, which is hydrophilic fumed silica, available in the market with the trademark name ‘Aerosil 200’. Although the primary size of nanosilica is 12 nm, due to the strong interparticle forces, these particles tend to aggregate to form agglomerates. The agglomeration phenomenon absolutely changes the fluidization dynamics of the nanosilica bed since the vertical balancing forces, such as drag and gravitational force, act on the agglomerates rather than fine particles. The size analysis of nanosilica reported in Figure 2 shows a size distribution varying from 10 to 200 µm and the Sauter mean diameter of 20 µm due to the agglomeration phenomenon [51].

Two different types of external particles were used in the experiments to alter the dynamics of the nanosilica bed. Both particles were inert silica of different size ranges. The first external particles were named as ‘S-A’, which constituted particles with a size ranging from 38 to 75 µm and a Sauter mean diameter of 56.5 µm, while the second external particles were termed as ‘S-B’, with particles ranging from 125 to 300 µm and a Sauter mean diameter of 212.5 µm. The size analysis of both S-A and S-B external particles is reported in Figure 2, while the physical properties are reported in Table 2. The size range of S-A external particles classifies it in Geldart group A and S-B in Geldart group B in Geldart’s chart.

### 2.4. Methodology

The experimental procedure involves three sections. Firstly, the unassisted fluidization of nanosilica was performed up to the air superficial velocity of 100 mm/s, followed by the defluidization of the bed, during which the airflow rate was gradually reduced until complete rest.

In the second set of experiments, the S-A external particles were added to the nanosilica bed and multiple runs were performed by varying the volumetric fraction of S-A particles at 2%, 4% and 8%, respectively. In the third part of the experiments, the S-B particles were used with nanosilica particles at the volumetric fraction of 2%, 4% and 8%, respectively

Before beginning the second and third set of experiments, the mixture of nanosilica and external particles was thoroughly homogenized by the air flow and the bed was allowed to settle overnight. Thereafter, the fluidization and defluidization experiments were conducted.

## 3. Result and Discussion

**Figure 3:**
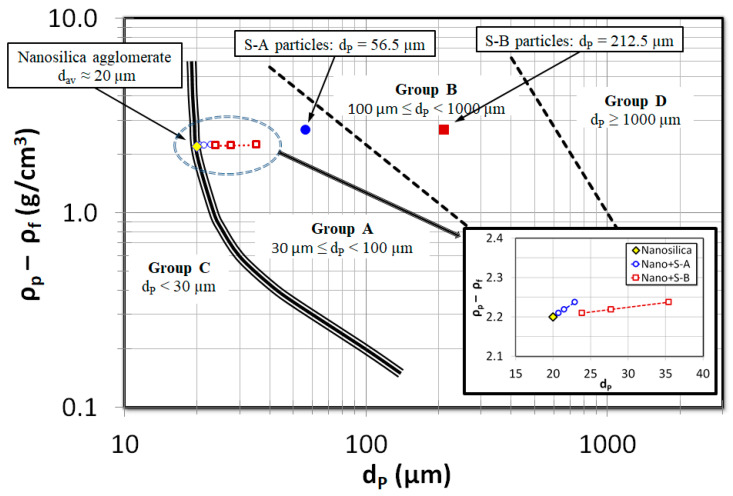
Geldart’s classification displaying position of nanosilica mixed with sand particles at compositions of 2 vol%, 4 vol% and 8 vol%.

**Figure 4:**
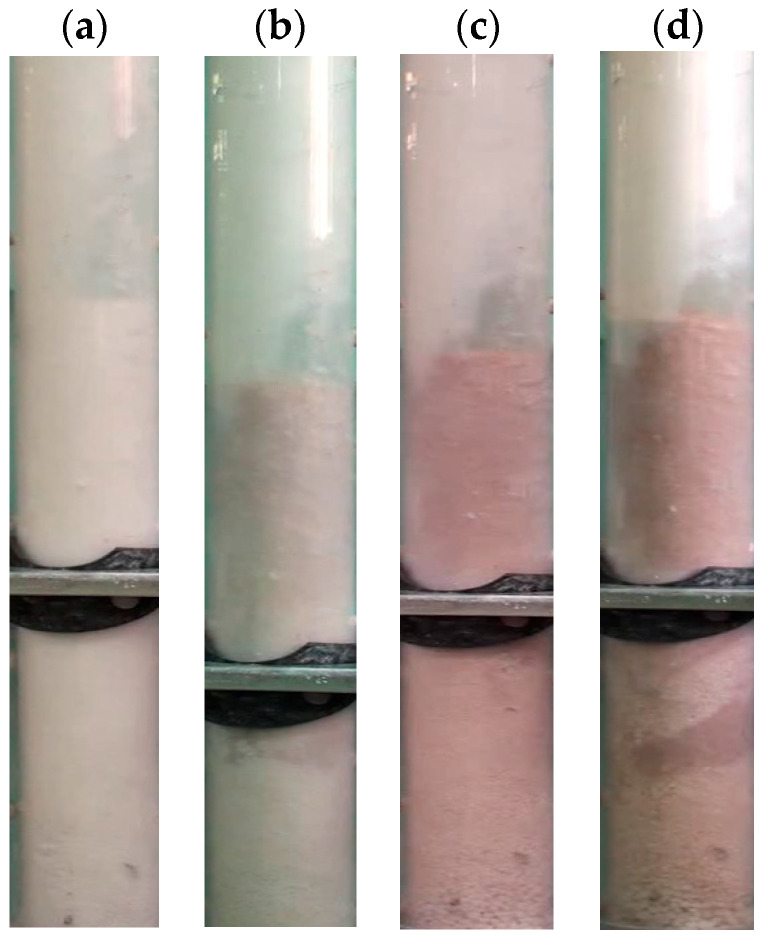
Digital images of the fluidized bed at incipient fluidization with different proportions of external particles S-A; (**a**) 0 vol%; (**b**) 2 vol%; (**c**) 4 vol%; (**d**) 8 vol%.

**Figure 5:**
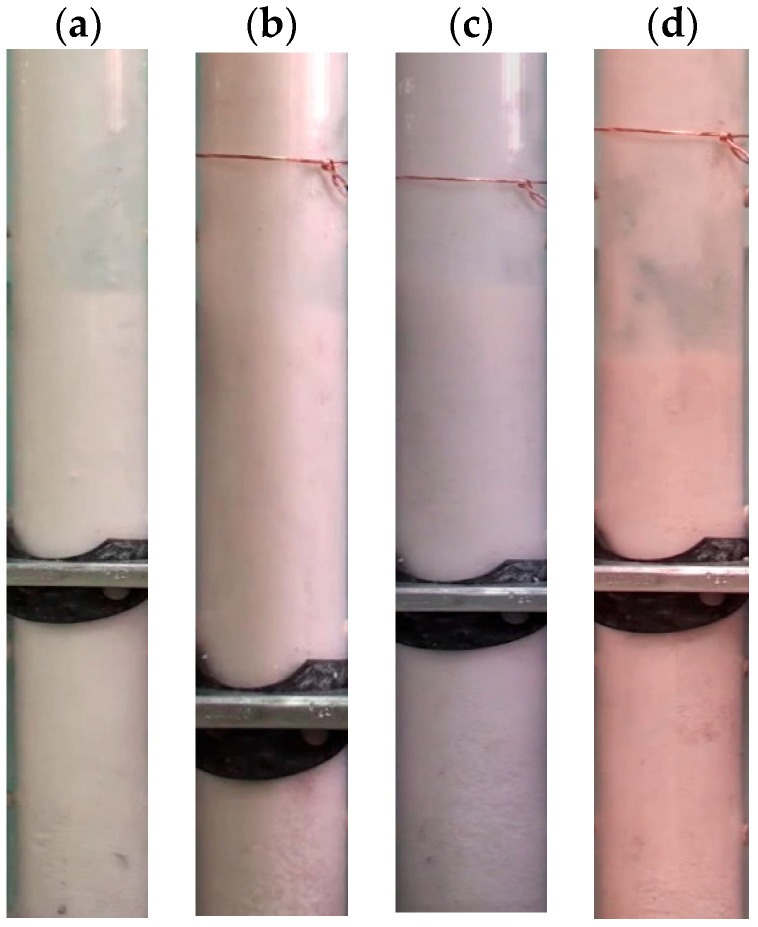
Digital images of the fluidized bed at incipient fluidization with different proportions of external particles S-B; (**a**) 0 vol%; (**b**) 2 vol%; (**c**) 4 vol%; (**d**) 8 vol%.

Figure 3 presents the Geldart classification of powders, with the average sizes of the nanosilica agglomerates and the external particles used for premixing (Group A and Group B) marked on the diagram. As shown, the average size of the Group A particles is approximately three times larger than that of the nanosilica agglomerates, whereas the Group B particles are nearly 10 times larger. The size difference effect on the gravitational force acting on the particles is substantially amplified due to the cubic dependence of the gravitational force on particle diameter. Furthermore, an inset illustrates how the mean particle size varies within the mixed beds at different premixing proportions. It is important to note that the skeletal densities of the external particles and the nanosilica agglomerates are comparable; thus, the observed variation in the mixed bed properties primarily arises from differences in particle size rather than density effects. Figure 4 displays the digital images of the nanosilica bed mixed with external particles S-A taken from the video (Supplementary Materials) recordings during the experiments. The first image (Figure 4a) consists of only nanosilica without any external particles S-A. When observed closely, it was found that the particles are aggregated to different-sized agglomerates and the bed is segregated in multiple regions based on the particles’ agglomerates sizes. The region closer to the distributor consisted of large and rigid agglomerates. This region generally remained static even at high airflow rates of $2U_{mf}$. The air moved through channels and voids at low velocities. However, few bubbles were visible at higher airflow rates. The middle region was a fluffy layer with soft agglomerates. The air bubble movement was prominent here. At high flow rates, this region also fluidized with the upper region. The uppermost layer comprised fine nanosilica particles, where the air bubbles erupted vigorously, generating high turbulence in the region. The bubbling intensity increased with the airflow rate, creating further turbulence in this bed region. Also, the region fluidized earlier than the incipient fluidization. External particles S-A were mixed subsequently in the bed in Figure 4b–d in a ratio of 2 vol%, 4 vol% and 8 vol%, respectively. As the images reveal, the particles are completely mixed throughout the bed; however, on closer observation, it was seen that the external particles were prominent in the upper and middle regions, while the coarse particles have not uniformly reached the lower region. The nanosilica bed in Figure 5b–d is mixed with external particles S-B in the ratio of 2 vol%, 4 vol% and 8 vol%. Here, the mixing is less notable between the particles in the upper region. The particles were dominant in the lower bed layer. In another work, we have calculated the region-wise particles’ agglomerate diameter in the nanosilica bed [52]. The average diameter was found to be 15–30 μm in the upper region, while the average diameter was 40–90 μm in the mid region and 100–250 μm in the lower region. The size range of external particles S-A, with Sauter diameter of 56.5 μm, is similar to the particles’ diameter in the upper and middle region, while the size range of external particles S-B (Sauter diameter: 212.5 μm) is similar to the lower region’s agglomerate diameter as compared to the nanosilica bed. The similar sized particles mixed well in the respective regions and, hence, the external particles S-A were notable in the upper region and middle region, and S-B in the lower region. Moreover, the phenomenon was further aided by gravity, which plunged the larger external particles S-B to the lower region. However, the small particles S-A prevailed in the upper regions due to the comparatively lesser impact of gravity.

**Figure 6:**
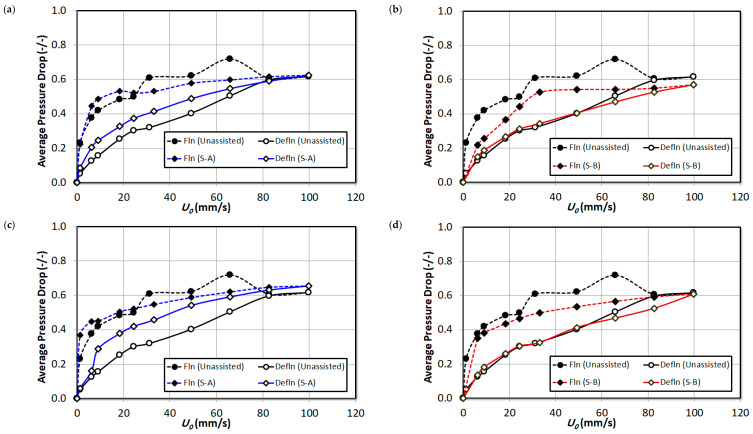

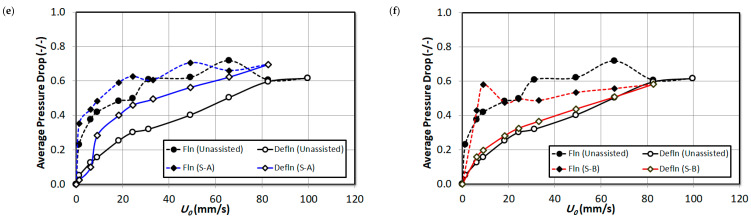
Comparison of overall pressure drop of unassisted bed with the external particle mixed bed at varying air superficial velocity; (**a**) 2 vol% S-A; (**b**) 2 vol% S-B; (**c**) 4 vol% S-A; (**d**) 4 vol% S-B; (**e**) 8 vol% S-A; (**f**) 8 vol% S-B.

Figure 6 reports the overall pressure drop during fluidization and defluidization with changing fluid velocities for unassisted run and the runs with external particles S-A and external particles S-B mixed at proportions of 2 vol%, 4 vol% and 8 vol%, respectively. The hysteresis phenomenon, which is prominent during the unassisted runs, has subdued with the addition of both external particles S-A and S-B. However, the effect of type and increment in external particles’ volumes is indeterminate here. The hysteresis phenomenon is caused by the indifference in fluidization and defluidization process due to persistent bed non-homogeneities during fluidization, while the bed attains a certain degree of homogeneity during defluidization. The fluidization index, which is often described as a normalized pressure drop, can effectively infer the fluidization behavior of the fluidized bed particles.

The fluidization index can be calculated as follows:

(1) FI=ΔP¯mgA

where $\bar{\Delta P}$ is the average pressure drop, m is the mass of the particles in the bed, and A is the cross-sectional area of the bed. The FI=1 indicates that the bed exhibits good fluidization quality. The bed is completely fluidized in the fluid and the overall pressure drop across the fluidized bed is equal to the effective bed weight. A low FI value signifies a partial fluidized bed with gas channeling and bypassing. The minimum fluidization velocity ($U_{mf}$) and fluidization index (FI) can be calculated from the overall pressure drop during fluidization. The calculated $U_{mf}$ and FI values are reported in Table 3. The $U_{mf}$ has significantly reduced by the addition of the external particles. Although, there is no apparent trend of the effect of addition of S-A external particle; however, the $U_{mf}$ has decreased with the increase in composition of S-B external particles in the nanosilica bed. Nevertheless, the fluidization index has rather decreased by external particle premixing. The $U_{mf}$ and FI indicates that the nanosilica bed was partially fluidized due to the segregation of the bed as discussed earlier and the phenomenon further intensified with the external particle premixing.

**Figure 7:**
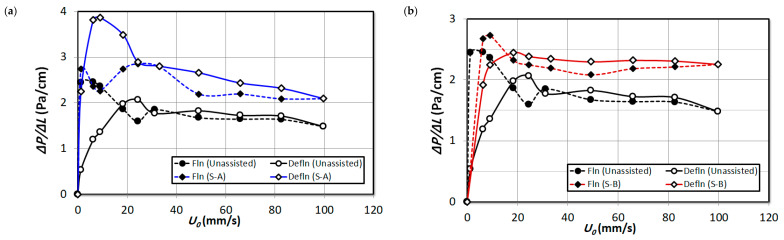

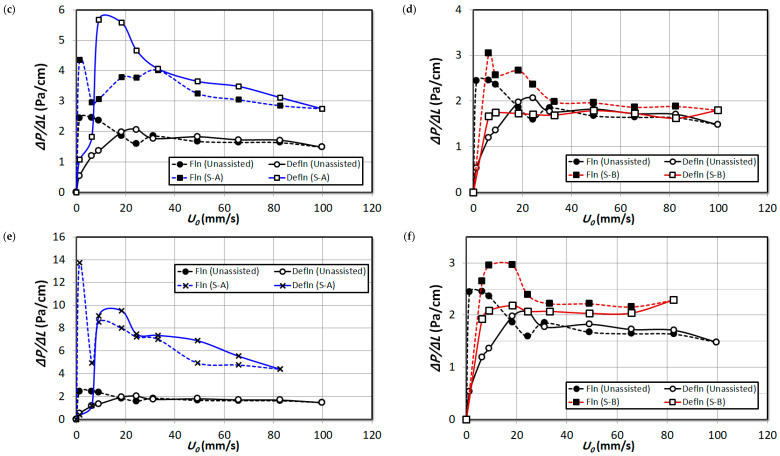
Comparison of local pressure drop in upper region (${∆P}_{4}$) of unassisted bed with the external particle mixed bed at varying air superficial velocity; (**a**) 2 vol% S-A; (**b**) 2 vol% S-B; (**c**) 4 vol% S-A; (**d**) 4 vol% S-B; (**e**) 8 vol% S-A; (**f**) 8 vol% S-B.

In order to obtain a detailed insight into the bed dynamics, the pressure drop transients recorded for different bed regions are analyzed separately in this section. The local pressure drop is normalized by the pressure tap distance so that an equitable comparison is obtained. Figure 7 illustrates the effect of increasing the proportion of external particles S-A and S-B in the upper bed region $\left(\Delta P_{4}\right)$ of the nanosilica bed. Fine particles exist in this region and there is no hysteresis visible for unassisted fluidization runs. Perhaps the addition of S-A external particles has marginally increased the hysteresis in this region. This is due to the significant presence of S-A external particles in the region, which have different size range. A similar observation was established in the bed images in Figure 4. It must be noted that the Sauter mean diameter of nanosilica agglomerates in this region is 22.5 μm, while the Sauter mean diameter of S-A particles is 56.5 μm, which resulted in uneven size; hence, they developed hysteresis. The hysteresis has reduced with the addition of 2 vol% S-B external particles in Figure 7b, which indicates that the fine particles have increased in the bed due to segregation and deagglomeration in the lower regions. However, further addition of S-B particles resulted in a marginal hysteresis, possibly due to the addition of some external particles in the region due to increased external particles composition in the bed. Comparing the beds during the fluidized state with 2 vol% addition of particle premixing in Figure 7a,b, it can be seen that the bed weight has slightly increased, in fact higher in Figure 7a, which corroborates the fact that the upper region is composed of significant volume of S-A external particles in the region. However, in Figure 7a, the pressure drop steadily decreased with the increase in the fluid flow rate. This phenomenon could be due to two reasons: the segregation of external particles occur, which settles down in the lower regions and due to the increase in fines in the upper bed region. In contrast, the pressure drop remains steady in Figure 7b for the case of S-B particle premixing. Increasing the proportion of external particles S-A in the bed in Figure 7c,e resulted in a further increase in pressure drop of the region. The decrease in pressure drop with the increase in fluid flow rate is steeper with the increase in volume of external particles S-A. However, the pressure drop is similar to the unassisted fluidization for the case of S-B particle premixing in Figure 7d,f, which clearly indicates although there could be possible addition of fines in the upper region; however, the composition of the S-B particles in the upper bed region was insubstantial, rather present in the lower bed regions only as it was observed in the bed images in Figure 5.

**Figure 8:**
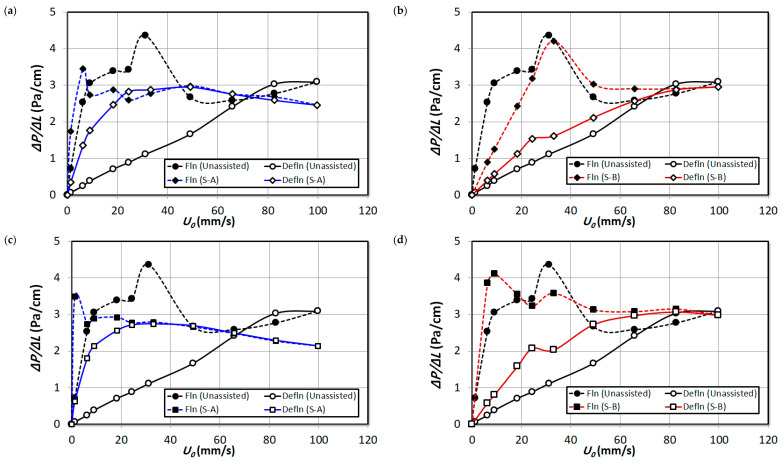

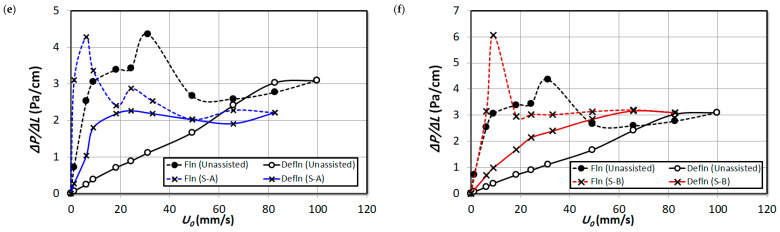
Comparison of local pressure drop in upper region (${∆P}_{3}$) of unassisted bed with the external particle mixed bed at varying air superficial velocity; (**a**) 2 vol% S-A; (**b**) 2 vol% S-B; (**c**) 4 vol% S-A; (**d**) 4 vol% S-B; (**e**) 8 vol% S-A; (**f**) 8 vol% S-B.

Figure 8 reports the pressure drop in the upper-middle region $\left(\Delta P_{3}\right)$ of the bed. For the case of unassisted fluidization, high hysteresis is visible here, considering the fact that the middle region comprises uneven sized fluffy agglomerates, which homogenized during defluidization, as observed visually. The hysteresis has significantly reduced with the addition of external particles, while the hysteresis reduction is much prominent in the case of S-A external particles due to the fact that the particles S-A existed predominantly in the upper regions of the bed. However, the increase in volume of external particles did not cause any change on the hysteresis phenomenon for both external particle premixing cases. Considering the incipient fluidization condition in Figure 8a, the $U_{mf}$ has significantly reduced with the addition of S-A external particles, which further reduced with the addition of 4 vol% and 8 vol% S-A external particles subsequently in Figure 8c,e. At high fluid flow rates during the fluidized bed condition, the decrease in pressure drop indicates the segregation of S-A particles from the nanosilica bed. An addition of 2 vol% of S-B particles did not induce any noticeable effect on the $U_{mf}$ as seen in Figure 8b. However, further addition of particles decreased the $U_{mf}$ in Figure 8d,f; however, the magnitude of the decrease in $U_{mf}$ is less than the S-A particle premixing case. The foregoing discussion clearly indicates that the effect of S-A particles is significant in the upper-middle region while the effect of S-B particles is minimal comparatively.

**Figure 9:**
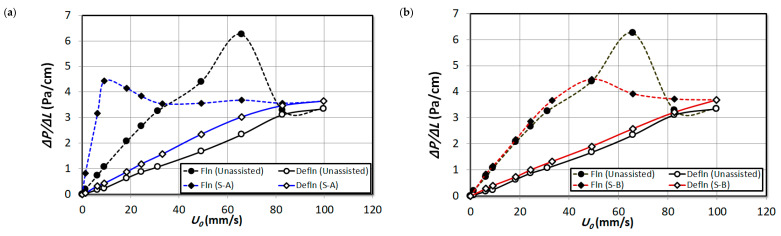

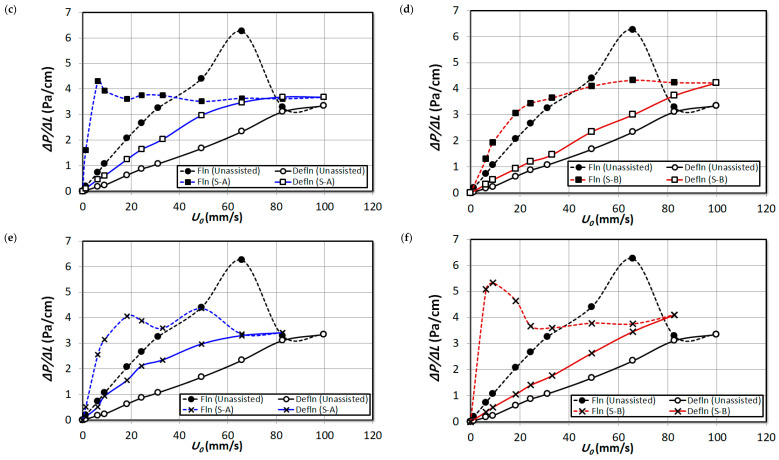
Comparison of local pressure drop in upper region (${∆P}_{2}$) of unassisted bed with the external particle mixed bed at varying air superficial velocity; (**a**) 2 vol% S-A; (**b**) 2 vol% S-B; (**c**) 4 vol% S-A; (**d**) 4 vol% S-B; (**e**) 8 vol% S-A; (**f**) 8 vol% S-B.

Figure 9 illustrates the pressure drop of the lower middle bed region ${(∆P}_{2})$. The $U_{mf}$ is higher here than the upper-middle region for unassisted fluidization due to the fact that the agglomerates size has increased while descending towards the bottom of the bed. While the hysteresis has lowered with the addition of both external particles S-A and S-B, respectively, the hysteresis is lower for S-B external particles than S-A comparatively. However, the incipient fluidization is achieved at low fluid rates for the S-A particle mixed bed in comparison to the S-B particle mixed bed. This could be due to the fact that the substantial presence of S-B particles in the particles raised the total bed weight which increased the $U_{mf}$. The $U_{mf}$ has decreased with the increase in volume of external particles for both case of S-A and S-B due to the increased deagglomeration phenomena, which indicates that both external particles were effective in improving the dynamics of this region.

**Figure 10:**
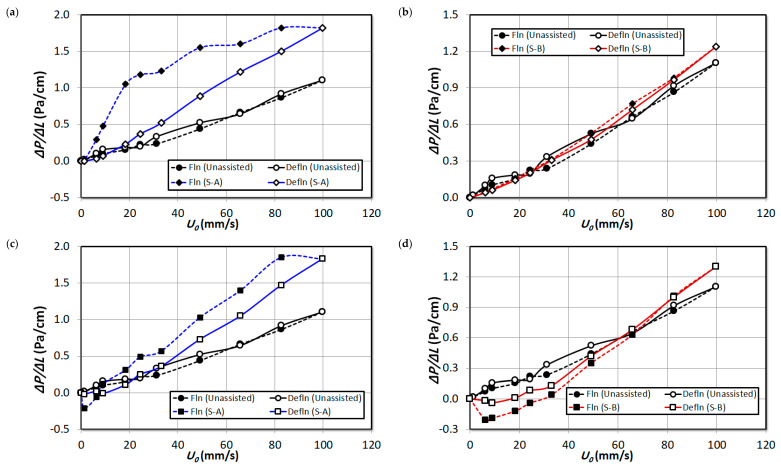

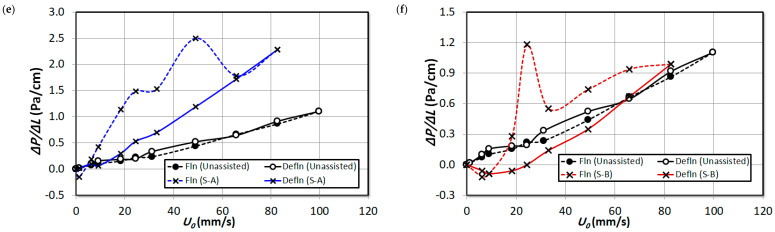
Comparison of local pressure drop in lower region (${∆P}_{1}$) of unassisted bed with the external particle mixed bed at varying air superficial velocity; (**a**) 2 vol% S-A; (**b**) 2 vol% S-B; (**c**) 4 vol% S-A; (**d**) 4 vol% S-B; (**e**) 8 vol% S-A; (**f**) 8 vol% S-B.

Figure 10 depicts the pressure at the lower bed region basically comprising hard and rigid agglomerates. The unassisted bed have not exhibited any hysteresis phenomenon in this region due to the segregation of particles’ agglomerates during the initial fluid flow rates of fluidization. There is no notable hysteresis for the case of S-B particle premixing in Figure 10b,d. It must be noted that the particle size of S-B particles is identical to the agglomerates’ size in the region and the bed was homogeneous with large sized particles and hence the hysteresis phenomena and the pressure profiles are similar to unassisted fluidization. This analysis also infers the fact that the effect of the 2 vol% and 4 vol% S-B particles’ effect was negligible in the lower region although the particles were present in high proportion in this region as observed in the bed images in Figure 5. However, increasing the volume to 8% displays significant hysteresis and bed compressed during fluidization causing negative pressure drop, which indicates that the higher proportion S-B particles have remarkably aided the deagglomeration phenomenon in the lower region Although there is hysteresis visible in Figure 10a,c when external particles S-A was added in the bed in proportions of 2 vol% and 4 vol%, and the hysteresis increased for the case of 8 vol% particle premixing. This could be due to the presence of S-A particles in scant quantity (not observed visually) in initial fluid flow rates during the fluidization, which segregated at higher fluid flow rates resulting in hysteresis.

**Figure 11:**
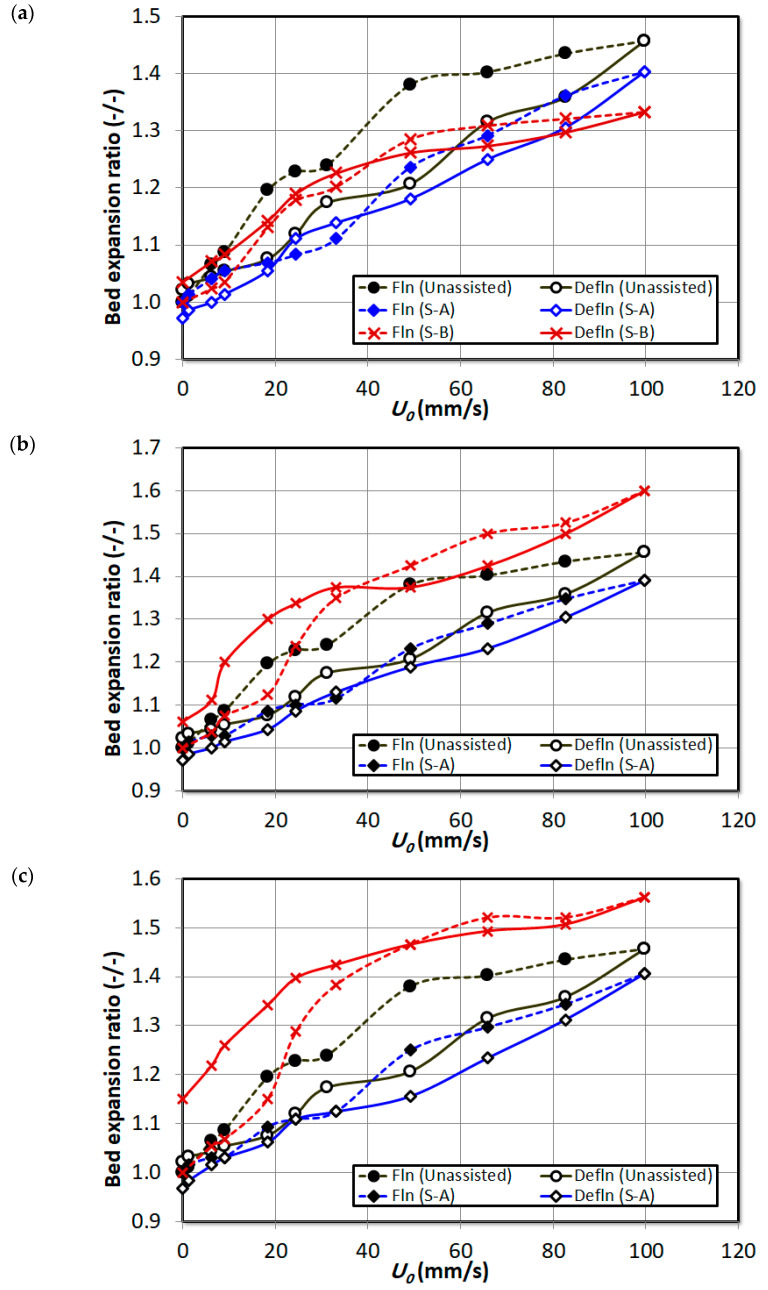
Bed expansion of unassisted bed with the external particle mixed bed at varying air superficial velocity; (**a**) 2 vol% particle premixing; (**b**) 4 vol% particle premixing; (**c**) 8 vol% particle premixing.

As seen earlier in overall pressure drop analysis in Figure 6, the overall bed hysteresis has decreased during bed expansion in Figure 11 with particle premixing. Another noteworthy feature here is that, with the increase in proportion of external particles S-B in the bed, the bed expansion has increased during defluidization. In fact, the bed expanded up to 15% of the original height during defluidization when 8 vol% of S-B particles were mixed in the bed.

Another aspect of comparison can be the volume change of mixing studies, which enables a comparison of bed void fraction of a pure component bed (nanosilica bed here) and multi component mixture bed. The bed void fraction can in turn provide insight of degree of particle premixing in the bed. The volume change of mixing during fluidization and defluidization for both cases of particle premixing was calculated using the following formula [49,53]:

(2) $$\chi =\frac{\Delta V}{V_{m}}\%$$

where ΔV is the volume change of mixing for two component mixture and $V_{m}$ is the specific volume of the mixture.

The ΔV can be calculated as

(3) $$\Delta V=V_{m}-\left(X_{1}V_{1}+X_{2}V_{2}\right)$$

where ${\;X}_{i}$ and $V_{i}$ are the initial volume fraction and specific volume of component i, respectively.

The specific volume V can be calculated as

(4) $$V=\frac{1}{1-\varepsilon }$$

where ‘ε’ is the void fraction of the bed.

**Figure 12:**
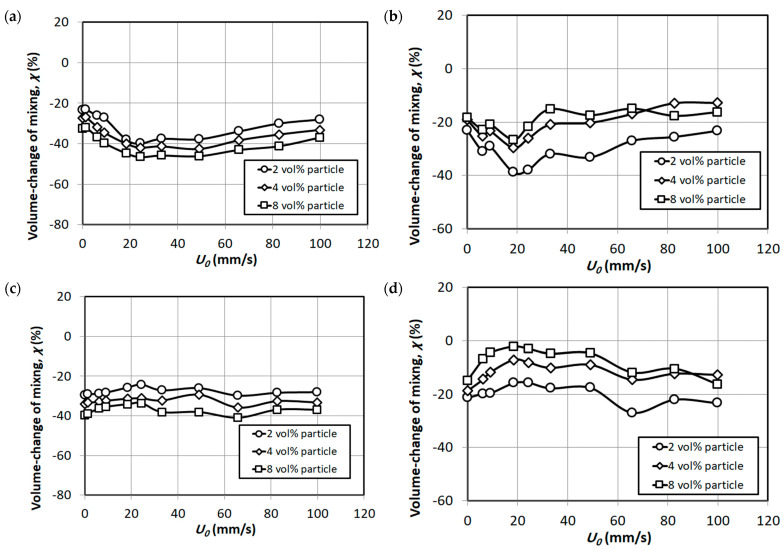
Volume change of mixing with the increase in external particles in the nanosilica bed; (**a**) fluidization (external particles S-A); (**b**) fluidization (external particles S-B); (**c**) defluidization (external particles S-A); (**d**) defluidization (external particles S-B).

A negative value of the volume change of mixing indicates that there is a contraction of the volume of the mixed bed. Figure 12 illustrates the volume change of mixing during fluidization and defluidization. The negative values indicate the degree of mixing of nanosilica and external particles in the bed. The negative values are apparent at zero flow rate due to the reason that the bed was homogenized before beginning the experiments in all cases. The higher negative values for S-A external particles signifies higher mixing of S-A particles and nanosilica in the bed in comparison to S-B external particles. As seen in Figure 12a, increasing the proportion of S-A external particles decreased the volume, which indicates that the particles were well mixed in the bed at all proportions. However, increasing the fluid flow rate resulted in segregation of particles for the case of S-B external particles in Figure 12c especially after incipient fluidization, the segregation is more prominent with the increase in volume of S-B external particles. It must be noted that as discussed earlier, the S-B particles were particularly prevalent in the lower sections of the bed. The segregation continued during defluidization as well, as seen in Figure 12d.

## 4. Conclusions

This study systematically demonstrates that the fluidization behavior of ultrafine nanosilica can be significantly enhanced by premixing with small quantities (<10 vol%) of inert Geldart Group A (S-A) and Group B (S-B) silica particles. A detailed region-wise analysis revealed a clear vertical segregation pattern within the fluidized bed: the finer S-A particles were predominantly prevalent and effective in the upper and upper-middle regions, while the coarser S-B particles were more prominent and impactful in the middle and lower bed regions.

Overall, premixing with both S-A and S-B particles led to a significant reduction in the minimum fluidization velocity ($U_{mf}$) and a decrease in the overall fluidization hysteresis. However, the influence on hysteresis exhibited regional variations. While the presence of S-A particles increased hysteresis in the upper region, it considerably decreased hysteresis in the upper-middle region, where the $U_{mf}$ also decreased with increasing S-A particle composition. Notably, the lower bed region, characterized by rigid agglomerates, remained largely unaffected by particle premixing, with the exception of an 8 vol% S-B particle addition, which resulted in considerable deagglomeration.

A key observation was the significant volumetric contraction (up to 40%) of the premixed nanosilica bed, even at low premixing proportions. This contraction, indicative of the disruption of inter-agglomerate force equilibrium and improved packing, was further corroborated by negative values for the volume change of mixing during both fluidization and defluidization phases. Furthermore, increasing the proportion of S-B particles in the bed led to an improved overall bed expansion during defluidization.

In summary, this work confirms that low-percentage particle premixing is a viable and low-energy assisted fluidization technique for improving the hydrodynamic behavior of cohesive nanosilica powders. The region-specific enhancements observed highlight the critical importance of carefully selecting external particle properties (such as size) to match the local characteristics within the nanosilica bed for targeted improvements. These insights provide a valuable basis for optimizing the design and operation of industrial fluidized beds for nanoparticle processing. Future research could beneficially explore the synergistic effects of combining this premixing strategy with other fluidization assistance methods.

## Figures and Tables

**Table 1:** Pressure tap positions used to record region-wise pressure transients.

Pressure Drop	Pressure Tap Positions (from the Distributor)	Bed Region
${∆P}_{g}$	0.05 m–open	Overall
${∆P}_{4}$	0.3 m–open	Upper
${∆P}_{3}$	0.2 m–0.3 m	Upper–middle
${∆P}_{2}$	0.1 m–0.2 m	Lower–middle
${∆P}_{1}$	0.05 m–0.1 m	Lower

**Table 2:** Physical properties of the solid particles used in the experiments in this work.

Particles’ Name	Chemical Formula	Primary Size	Sauter Mean Diameter (μm)	True Density kgm3
Nanosilica	SiO_2_	12 nm	20 μm	2200
S-A	SiO_2_	38–75 μm	56.5 μm	2664
S-B	SiO_2_	125–300 μm	212.5 μm	2664

**Table 3:** Minimum fluidization velocity and fluidization index calculated from Figure 6.

	Vol. %	$U_{mf}$ (mm/s)	*F.I.* (Dimensionless)
Unassisted		33.0	0.62
S-A	2%	22.0	0.57
	4%	19.0	0.58
	8%	26.0	0.65
S-B	2%	32.5	0.55
	4%	22.0	0.56
	8%	19.0	0.54

## Data Availability

Data are contained within the article.

## Supplementary Materials

The following supporting information can be downloaded at: https://www.mdpi.com/article/10.3390/nano15110822/s1, Video S1: nanosilica bed mixed with external particles S-A.

## Nomenclature

A	cross-sectional area of the bed
m	mass of the particles in the bed
$\bar{\Delta P}$	average pressure drop (Pa)
ΔV	volume change of mixing for two component mixture (m^3^/kg)
$V_{m}$	specific volume of the mixture (m^3^/kg)
$V_{i}$	specific volume of component i (m^3^/kg)
$X_{i}$	initial volume fraction (-/-)
Greek symbols	
ε	bed porosity (-/-)
$\rho_{f}$	air density (kg/m^3^)
$\rho_{p}$	true solid density (kg/m^3^)
